# Killing Effects of an Isolated *Serratia marcescens* KH-001 on *Diaphorina citri* via Lowering the Endosymbiont Numbers

**DOI:** 10.3389/fmicb.2018.00860

**Published:** 2018-05-01

**Authors:** Wei Hu, Fan Kuang, Zhanjun Lu, Ning Zhang, Tingtao Chen

**Affiliations:** ^1^National Navel Orange Engineering Research Center, College of Life and Environmental Sciences, Gannan Normal University, Ganzhou, China; ^2^Institute of Translational Medicine, Nanchang University, Nanchang, China

**Keywords:** *Serratia marcescens*, *Diaphorina citri*, OTU, high-throughput sequencing, Huanglongbing (HLB)

## Abstract

Huanglongbing (HLB) is the most devastating citrus disease worldwide, and suppression of the Asian citrus psyllid (*Diaphorina citri*) is regarded as an effective method to inhibit the spread of HLB. In this study, we isolated a strain named as *Serratia marcescens* KH-001 from *D. citri* nymphs suffering from disease, and evaluated its killing effect on *D. citri* via toxicity test and effect on microbial community in *D. citri* using high-throughput sequencing. Our results indicated that *S. marcescens* KH-001 could effectively kill 83% of *D. citri* nymphs, while the fermentation products of *S. marcescens* KH-001 only killed 40% of the *D. citri*nymphs. High-throughput sequencing results indicated that the *S. marcescens* KH-001 increased the OTU numbers from 62.5 (PBS buffer) to 81.5, while significantly lowered the Shannon index compared with *Escherichia coli* DH5α (group E) (*p* < 0.05). OTU analysis showed that the *S. marcescens* KH-001 had significantly reduced the relative abundance of endosymbionts *Wolbachia*, *Profftella*, and *Carsonella* in group S compared with that in other groups (*p* < 0.05). Therefore, the direct killing effect of the fermentation products of *S. marcescens* KH-001 and the indirect effect via reducing the numbers of endosymbionts (*Wolbachia*, *Profftella*, and *Carsonella*) of *D. citri* endow *S. marcescens* KH-001 a sound killing effect on *D. citri*. Further work need to do before this strain is used as a sound biological control agents.

## Introduction

Huanglongbing (HLB, also known as citrus greening) is a devastating citrus disease caused by *Candidatus* Liberibacter spp., which is first reported in China in 1943 and has been reported in at least 40 citrus producing countries (Bové, 2006). Till now, there is no cure for HLB once the trees get infected, and traditional HLB managements rely on psyllid control, aggressive removal of infected trees and planting of disease-free nursery trees (Grafton-Cardwell et al., 2013; Alvarez et al., 2016). However, growers are reluctant to follow these traditional HLB managements because it is costly and difficult to replant trees and get them into production, therefore most growers choose not to remove diseased trees if they remain productive (Hall and Gottwald, 2011).

Three different species of *Candidatus* Liberibacter have been identified, e.g., *Candidatus* Liberibacter asiaticus (Las), *Candidatus* Liberibacter africanus, and *Candidatus* Liberibacter americanus. Among them, Las is the most widely distributed HLB pathogen and is vectored by Asian citrus psyllid (ACP) *Diaphorina citri* Kuwayama (Hemiptera: Liviidae) (Bové, 2006). *D. citri* depends heavily on young citrus flush for survival and reproduction, and the feeding and reproductive behavior of *D. citri* plays an important role in the spread of Las. *D. citri* adults prefer emerging plant tissues for oviposition (Halbert and Manjunath, 2004), which make young trees particularly prone to Las infection. Moreover, the volatile methyl salicylate compound released by the infected trees attracts *D. citri* adults (Mann et al., 2012). Therefore, suppression or reduction of *D. citri* has been the primary method to inhibit the spread of HLB (Saha et al., 2017).

Biological control is one of the most promising approaches to control pests (Peshin et al., 2009; Alvarez et al., 2016), which offers an alternative to chemical pest control for its high selectivity, neglected effect on environment (Abdel-Baky and Abdel-Salam, 2003). Here, we isolated a bacterium from *D. citri* nymphs suffered from disease in citrus orchard (Gannan Normal University, Ganzhou City, Jiangxi Province), which was identified as *Serratia marcescens* using biochemical identification and molecular biological identification. Then this stain was named as *S. marcescens* KH-001 and stored at China Center for Type Culture Collection (Registration No. CCTCC M 2017465).

As we known, the microbiome hosted in *D. citri* played an important role on host growth, development, spawning (Coletta-Filho et al., 2005). As a powerful tool, high-throughput sequencing technology can detect almost all the DNA signatures of microbes within specific environments, even the bacteria with a low numbers or in the dormant metabolic state (Kobayashi et al., 2006; Wang et al., 2011; Zhang et al., 2012), and the high distinguish ability and sensitivity of the high-throughput sequencing provided us the actual microbial composition in *D. citri*. Therefore, our group investigated the killing effects of fermented supernatant of *S. marcescens* KH-001, *Escherichia coli* DH5α and *S. marcescens* KH-001 on *D. citri*, and we also studied their effects on the parasitic microbial diversity in *D. citri* using high-throughput sequencing method, to explore the possible killing mechanism of *S. marcescens* KH-001 on *D. citri*.

## Materials and Methods

### Bacteria Isolation and Identification

The *D. citri* nymphs suffered from disease in citrus orchard (Gannan Normal University, Ganzhou City, Jiangxi Province) were sampled, then they received surface sterilization using 75% ethanol for 5 min, and washed for three times with sterile water in a clean bench. Then washed *D. citri* were placed onto the LB solid medium, and the *D. citri* were broken down using transferring loop and cultured for 24 h at 28°C. Then the red colonies were picked out and purified using agar-streak method. Forty five strains were randomly selected and identified using biochemical identification and molecular biological identification, and a strain of *S. marcescens* were finally selected based on its sound killing effect on *D. citri* nymphs, which was named as *S. marcescens* KH-001 and stored at China Center for Type Culture Collection (Registration No. CCTCC M 2017465).

### Virulence of the *S. marcescens* KH-001 on *D. citri*

The *D. citri* Kuwayama (Hemiptera: Liviidae) were collected from citrus orchard (Tandong Town, Ganzhou City, Jiangxi Province 25°47′38′′ N, 114°25′4′′ E), seedling at 27 ± 1°C, 68% RH, and 14:10 h L:D photoperiod. The *D. citri* was maintained on *Murraya exotica* L. and was consecutive propagated for 20 generations before experiment.

*Serratia marcescens* KH-001 and *E. coli* DH5α were cultured in 5 ml LB medium and cultured for 6 h at 28°C. Then 1% of the above-mentioned mixture was inoculated into 5 ml LB medium and cultured for another 12 h. The cultures were centrifuged at 4500 ×*g* for 10 min at 4°C, and were resuspended using PBS buffer. Then *D. citri* (Fifth instar nymphs) were randomly divided into four groups: C, treated with PBS (*N* = 40); A, treated with the fermented supernatant of *S. marcescens* KH-001 (*N* = 40); E, treated with the *E. coli* DH5α containing in PBS buffer (*N* = 40); S, treated with the *S. marcescens* KH-001 containing in PBS buffer (*N* = 40). For all groups, 0.4 μl liquid were dropped onto the back of *D. citri*, and all *D. citri* were covered with cling film, and placed in the incubator (27 ± 1°C, 14 h light:10 h dark), and the survival rate of *D. citri* were recorded every 3 h.

### Extraction of Genome DNA and High-Throughput Sequencing

As it is difficult to isolated sufficient DNAs from a single *D. citri*, so 10 *D. citri* were mixed as one sample for the DNA extraction. The genomic DNAs of each sample were extracted by the TIANamp Genomic DNA kit (TIANGEN) (Yu et al., 2015). The extracted DNAs were used as templates, and the universal primer pair 338F/806R with the respective barcode for ease of identification were used to amplify the V3–V4 region of 16S ribosomal (r)RNA genes of all samples (GenBank Accession No. SUB3652522). PCR reactions, pyrosequencing of the PCR amplicons and quality control of raw data were performed as described previously (Xu et al., 2015)

### Bioinformatics and Multivariate Statistics

To eliminate the low-quality sequences, PyroNoise algorithm in Mothur (version 1.33.3) were used (Schloss et al., 2009), and the Quantitative Insights Into Microbial Ecology (QIIME) platform (version 1.8.0) was implemented for Bioinformatics analysis (Caporaso et al., 2010). Briefly, 16S rRNA operational taxonomic units (OTUs) were clustered using an open-reference OTU picking protocol based on 97% nucleotide similarity with the UCLUST algorithm (Davenport et al., 2014), and ChimeraSlayer was applied to remove chimeric sequences (Haas et al., 2011). The relative abundance of each OTU was determined as a proportion of the sum of sequences for every sample, taxonomic relative abundance profiles were generated based on OTU annotation, and the microbial community structure was evaluated by biodiversity. Shannon index, phylogenetic diversity, chao1 index and the observed number of species were used to evaluate α diversity, and the weighted and unweighted UniFrac distances were used to evaluate β diversity using QIIME pipeline.

### Statistical Analysis

The statistical significance of the relationships among different groups was evaluated by Wilcoxon and Kruskal–Wallis tests and Spearman’s rank correlation. The data were presented as mean ± SD, and *p* < 0.05 or *p* < 0.01 was considered statistically significant (Lu et al., 2014).

## Results

### Bacterial Isolation and Its Virulence on *D. citri* Nymphs

To isolate the potential bacteria killing *D. citri* nymphs, we sampled the *D. citr*i nymphs suffering disease and 45 bacteria were isolated from them. Finally, *S. marcescens* KH-001 were selected for our further evaluation.

**FIGURE 1:**
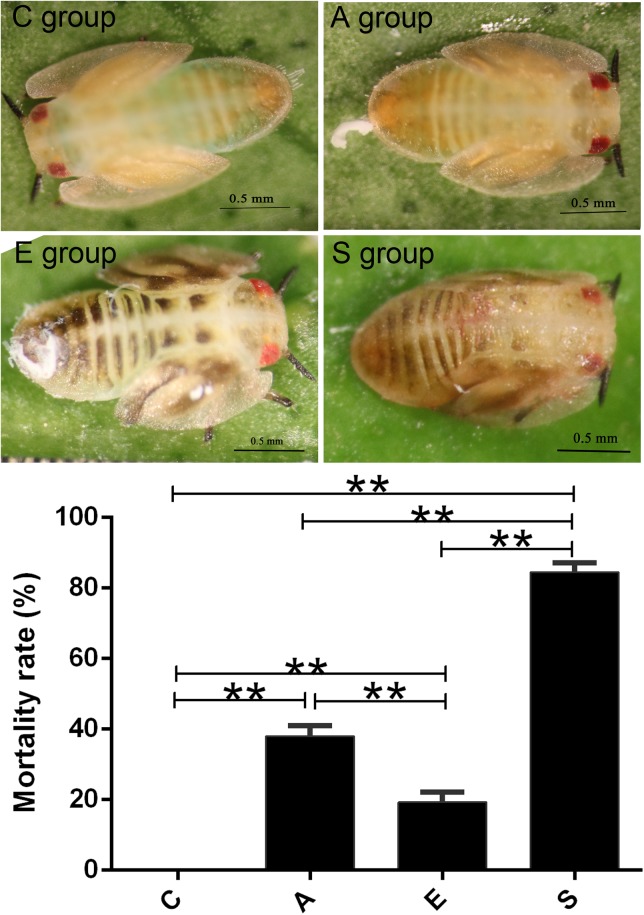
Killing effect of the PBS (C), Fermentation product of *Serratia marcescens* KH-001 (A), *Escherichia coli* DH5α (E), and *S. marcescens* (S) KH-001 on *Diaphorina citri* at 24 h. ^∗^*p* < 0.05; ^∗∗^*p* < 0.01.

Then we treated the artificial rearing *D. citri* nymphs using PBS buffer (group C), supernatant of *S. marcescens* KH-001 (group A), *E. coli* DH5α (group E), and *S. marcescens* KH-001 (group S). In **Figure 1**, our results indicated that no *D. citri* nymphs was dead in negative control group C, only 20% of *D. citri* nymphs were killed by the control strain of *E. coli* DH5α. Interestingly, the supernatant of *S. marcescens* KH-001 killed about 40% of *D. citri* nymphs, while *S. marcescens* KH-001 possessed the best killing rate of 83% in group S (*p* < 0.05).

### The OUT Analysis of Groups C, A, E, and S

**FIGURE 2:**
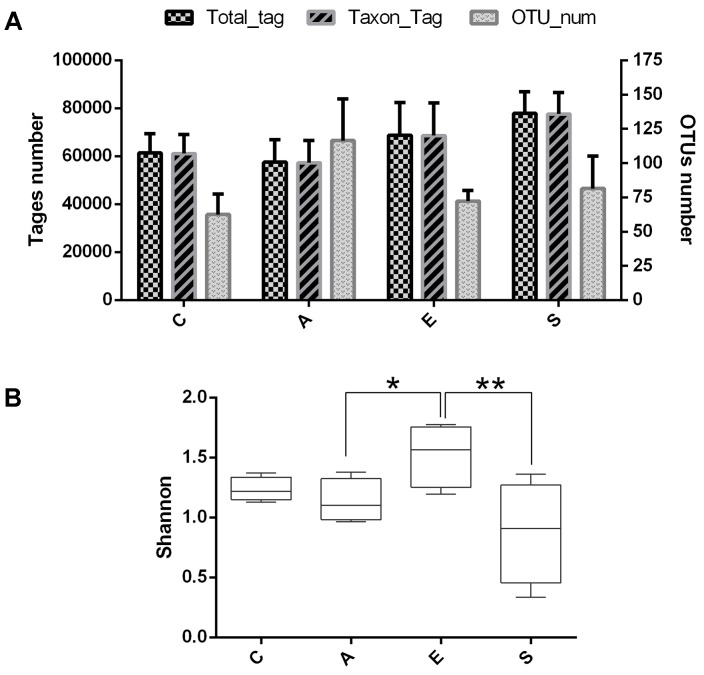
The OUT analysis of groups C, A, E, and S. **(A)** Number of total tags, taxon tags, and OTUs in groups C, A, E, and S. **(B)** The alpha-diversity distances calculated using phylotype relative abundance measurements among groups C, A, E, and S. C, PBS group; A, Fermentation product of *S. marcescens* KH-001 group; E, *E. coli* DH5α group; S*, S. marcescens* KH-001 group. ^∗^*p* < 0.05; ^∗∗^*p* < 0.01.

To evaluate the effects of fermented supernatant of *S. marcescens* KH-001, *E. coli* DH5α and *S. marcescens* KH-001 on microbial diversity in *D. citri*, 16S rRNA amplicon sequencing analysis was used to sequence the V3-V4 hypervariable region. All the effective tags of all samples were clustered and those sequences with over 97% similarity were considered as one OTU. In total, 1,062,236 usable raw sequences and 1,331 OTUs were obtained from all the samples with an average of 83.18 OTUs per group (**Figure 2A**). Moreover, the Shannon index indicated that the relative abundance of group E was higher than group A (*p* < 0.05), and the relative abundance of group S was significantly lower than that in group E (*p* < 0.01).

### The β Diversity of the Microbial Community in Groups C, A, E, and S

**FIGURE 3:**
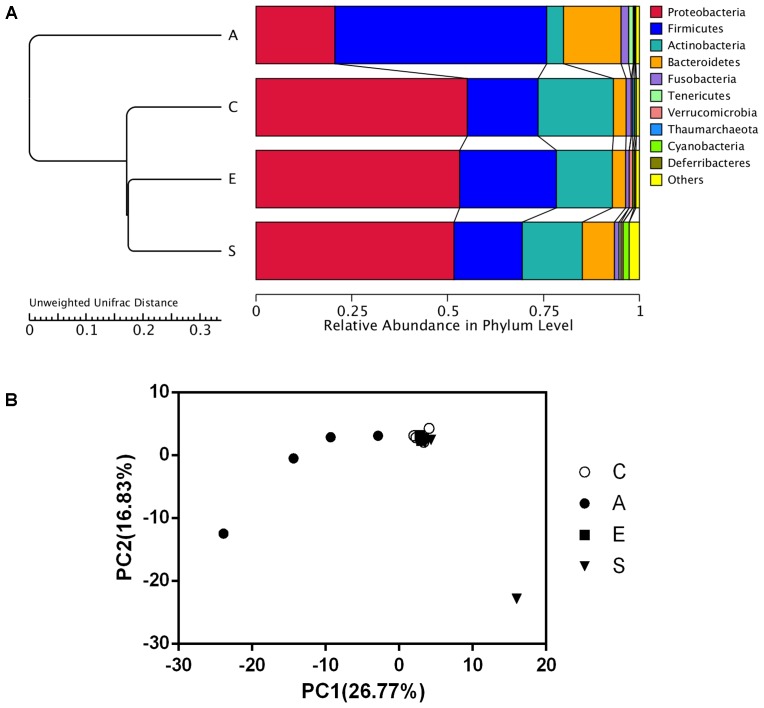
The UPGMA clustering tree Based on the Weighted UniFrac distance at phylum level **(A)** and the principle component analysis (PCA) of the microbial diversity **(B)** in groups C, A, E, and S. C, PBS group; A, Fermentation product of *S. marcescens* KH-001 group; E, *E. coli* DH5α group; S, *S. marcescens* KH-001 group.

As shown in **Figure 3A**, data of the top 10 microorganism populations was analyzed using unweighted Pair-group Method with Arithmetic Mean (UPGMA) to check the similarity between different groups (24). *Proteobacteria*, *Firmicutes*, *Actinobacteria*, and *Bacteroidetes* constituted four common dominant phyla and accounted for >90% of the total sequencing number in all groups. When treated with fermented supernatant of *S. marcescens* KH-001 (A), *E. coli* DH5α (E) and *S. marcescens* KH-001 (S), the relative abundance of *Firmicutes* and *Bacteroidetes* received an obvious reduction, while the abundance of *Proteobacteria* and *Actinobacteria* were greatly enhanced (**Figure 3A**). Moreover, the UPGMA and principal component analysis (PCA) results indicated that the microbial composition in groups C, E, and S possessed high similarity, while group A scattered away from groups C, E, and S (**Figure 3B**).

### The Specificity of Bacterial Communities in Groups C, A, E, and S

**FIGURE 4:**
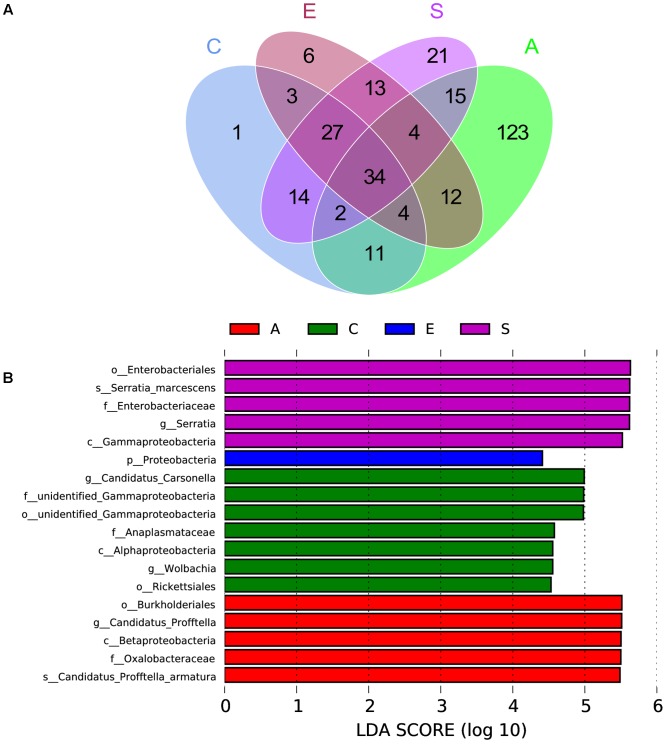
The scalar-Venn representation of shared genera among microbiome in groups C, A, E, and S **(A)** and the taxonomic representation of statistically significant differences among groups C, A, E, and S **(B)**. C, PBS group; A, Fermentation product of *S. marcescens* KH-001 group; E, *E. coli* DH5α group; S, *S. marcescens* KH-001 group.

The Venn figure reflecting the difference among groups C, A, E and S was shown in **Figure 4A**, there were 96, 205, 103, and 130 OTUs in groups C, A, E, and S, respectively. The comparison among all groups was done and only 34 common OTUs were detected. In addition, the statistically significant differences among groups C, A, E, and S indicated that the relative abundance of *Wolbachia*, *Anaplasmataceae*, *Alphaproteobacteria*, *Gammaproteobacteria*, *Rickettsiales*, *Carsonella* in C group was significantly higher than that in groups A, E, and S (**Figure 4B**, *p* < 0.05).

### The Relative Abundance of Bacteria Related to *D. citri*

**FIGURE 5:**
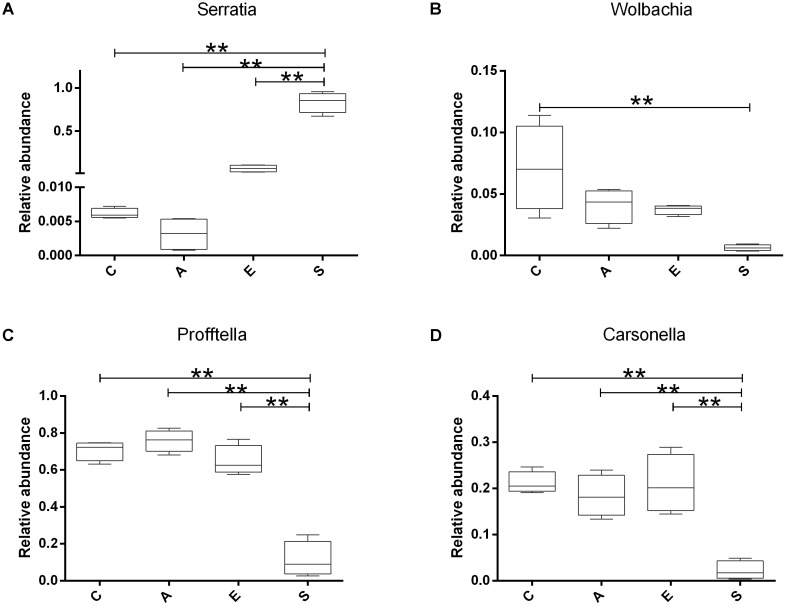
The relative abundance of *Serratia* **(A)**, *Wolbachia* **(B)**, *Profftella* **(C),** and *Carsonella* **(D)** in groups C, A, E, and S. C, PBS group; A, Fermentation product of *S. marcescens* KH-001 group; E, *E. coli* DH5α group; S, *S. marcescens* KH-001 group. ^∗^*p* < 0.05; ^∗∗^*p* < 0.01.

Based on the sequencing results, we compared the relative abundance of *Serratia*, *Wolbachia*, *Profftella*, and *Carsonella* in groups C, A, E, and S. The **Figure 5** showed that the *Serratia* in group S was significantly higher than that in groups C, A, and E (*p* < 0.01); the *Wolbachia* in group C was significantly higher than that in group S (*p* < 0.01); the *Profftella* and *Carsonella* in group S was significantly lower than that in groups C, A, and E (*p* < 0.01).

## Discussion

HBL were caused by the *Candidatus* Liberibacter spp. in phloem. It is estimated that the HBL occurred in more than 40 countries, and infected about billions of citrus plants, which caused 30–100% of the *Citrus* production (Crafts-Brandner, 2002; Bové, 2006; Iftikhar et al., 2016). In Florida, a production loss of $1.7 billion between 2006 and 2011 were observed, and consumers and producers lost an estimated $1 billion in the 2012–2013 season (Alvarez et al., 2016).

*Diaphorina citri* Kuwayama (Hemiptera: Liviidae) was the most important insect vector for HBL (Hall and Hentz, 2016; Saha et al., 2017). Numerous researches indicated that insect borne pathogen could affect the growth and development of media insects, e.g., the tomato yellow leaf curl virus could decrease the *Fecundity* and survival of *Bemisia tabaci*, and the *Alfalfa* mosaic virus could lower the survival rate and population growth rate of *Aphids* (Donaldson and Gratton, 2007). Therefore, our group sampled the *D. citri* nymphs suffered from disease in citrus orchard, and isolated a potential strain (*S. marcescens* KH-001) to kill the *D. citri* nymphs based on the previous report (Arp et al., 2017).

First of all, we compared the killing effect of supernatant of *S. marcescens* KH-001 (group A) and *S. marcescens* KH-001 (group S) on *D. citri* nymphs, and the PBS (group C), and *E. coli* DH5α (group E) were used as control groups. The virulence experiment indicated that *S. marcescens* KH-001 could effectively kill 83% of *D. citri* nymphs, while the killing rates were only 40 and 20% in group A (supernatant of *S. marcescens* KH-001) and group E (*E. coli* DH5α), indicating that the directly killing effect of the fermentation products of *S. marcescens* KH-001 was not the sole reason to kill *D. citri* nymphs (**Figure 1**).

A recent study indicated that *D. citri* experienced significant mortality when exposed to *S. marcescens* because of the lacking of antimicrobial peptides and the Imd pathway, revealing a reduced innate immune system defensed against Gram negative bacteria (Arp et al., 2017). However, no study is carried out to explore the relationship tween *S. marcescens* and the microbial diversity in *D. citri*. In nature, a surprisingly high number of endosymbionts harbored in the arthropods, and these endosymbionts had greatly influenced the nutrition, reproduction, evolution, and survival of their host (Zug and Hammerstein, 2012). Therefore, the high-throughput sequencing technology was used to investigate the effect of *S. marcescens* KH-001 on the microbial diversity hosting in *D. citri* nymphs.

Our results indicated that an average of 83.18 OTUs per group was obtained, the supernatant of *S. marcescens* KH-001 and *S. marcescens* KH-001 increased the OTU numbers in groups A and S from 62.5 (group C) to 116.5 and 81.5, respectively (**Figure 1**). For group E, though *E. coli* DH5α possessed little effect on the OTU numbers compared with group C, its Shannon index was significantly higher than that in groups A and S (*p* < 0.05). As we known, the Shannon index is an important indicator of bacterial diversity, and the higher microbial diversity is regarded as a key point for host to defense the alien invasions (Chen et al., 2011, 2012; Zhang et al., 2015; Erdman, 2016; Kakihana et al., 2016). Therefore, the significant reduction of Shannon index caused by *S. marcescens* KH-001 indicated that *S. marcescens* KH-001 could effectively kill *D. citri* nymphs via reducing the microbial diversity to weak their defense to external interference (**Figure 2**).

In addition, data of top 10 microorganism populations was analyzed using UPGMA to check the similarity among different groups (24). *Proteobacteria*, *Firmicutes*, *Actinobacteria*, and *Bacteroidetes* constituted four common dominant phyla and accounted for >90% of the total sequencing number in all groups. When treated with fermented supernatant of *S. marcescens* KH-001 (A), *E. coli* DH5α (E) and *S. marcescens* KH-001, the relative abundance of *Firmicutes* and *Bacteroidetes* received an obvious reduction, while the abundance of *Proteobacteria* and *Actinobacteria* were greatly enhanced (**Figure 3A**). Moreover, the UPGMA and PCA results indicated that the microbial composition in groups C, E, and S possessed high similarity, scattering away from group A (**Figure 3B**).

Next, we compared the OTUs in groups C, A, E, and S (**Figure 4**). The Venn results indicated there were 96, 205, 103, and 130 OTUs in groups C, A, E and S, and their common OTUs was only 34, and the unique OTUs specifically contained in groups C, A, E, and S were 1, 123, 6, and 21 (**Figure 4A**), and the relative number of *Wolbachia*, *Anaplasmataceae*, *Alphaproteobacteria*, *Gammaproteobacteria*, *Rickettsiales*, *Carsonella* in C group were significantly higher than that in other groups (*p* < 0.05) (**Figure 4B**).

In previous studies, researchers found that the endosymbionts of *Wolbachia*, *Profftella*, and *Carsonella* possessed high infection rate on *D. citri* (Guidolin and Cônsoli, 2013; Sun et al., 2014; Arp et al., 2017). Of which *Wolbachia* was regarded as one of the world’s most common parasitic microbes infected a high proportion of insects. As many as 25 to 70% of all insect species were estimated to be potential hosts of *Wolbachia*, and some host species could not reproduce, or even survive, without *Wolbachia* infection (Werren et al., 1995; Kozek et al., 2007). *Carsonella* was an endosymbiont was not only parasitic in its host insect, but also supplied the host with some essential amino acids (Thao et al., 2001; Tortora et al., 2007). *Profftella* was a *Betaproteobacterium*, could produce a defensive polyketide (diaphorin) and it could potential influence the biology of *D. citri* (Kondo et al., 2005; Fischer and Seitz, 2012). Therefore, we compared the relative abundance of *Serratia*, *Wolbachia*, *Profftella*, and *Carsonella* in groups C, A, E, and S (**Figure 5**). Our results indicated that the *Serratia* in group S was significantly higher than other groups (*p* < 0.01), and the few OTU numbers of *Serratia* in groups C, A, and E indicated that the *Serratia* was a common genius in *D. citri* nymphs (**Figure 5A**). Moreover, the addition of *S. marcescens* KH-001 had significantly reduced the endosymbionts of *Wolbachia*, *Profftella* and *Carsonella*, while the supernatant produced by the *S. marcescens* KH-001 posted little effect on these three genuses (**Figure 5**). Considering the important role of *Wolbachia*, *Profftella*, and *Carsonella* in the reproduce, development and survive of *D. citri*, we safely proposed that *S. marcescens* KH-001 might play its sound killing effect on *D. citri* via reducing the numbers of endosymbionts (*Wolbachia*, *Profftella*, and *Carsonella*) in *D. citri* (**Figures 1, 4, 5**).

In the present study, our group isolated a *S. marcescens* from *D. citr*i nymphs suffering disease, and our results indicated that this stain could effetely kill *D. citr*i via reducing the number of endosymbiosis, together with the direct killing effect of the supernatant (Arp et al., 2017). We first explored the potential relationship between the killing effect of *S. marcescens* KH-001 and the microbial diversity of endosymbionts in *D. citr*i, and provided basic data to use this strain as sound biological control agent. However, limited by the differences of host and geographical location, interactions between symbionts and HLB pathogen, much more work is need before this strain is applied in the biological control of the HBL via killing the insect vector of *D. citri*.

## Author Contributions

TC and NZ designed the experiment. WH, FK, and ZL performed the experiments. TC analyzed the data and wrote the manuscript. All authors discussed the results and commented on the manuscript.

## Conflict of Interest Statement

The authors declare that the research was conducted in the absence of any commercial or financial relationships that could be construed as a potential conflict of interest.
